# 2019 revised algorithm for the management of knee osteoarthritis: the Southeast Asian viewpoint

**DOI:** 10.1007/s40520-021-01834-x

**Published:** 2021-03-28

**Authors:** Swan Sim Yeap, Aree Tanavalee, Emmanuel C. Perez, Maw Pin Tan, Bernadette Heizel M. Reyes, Joon Kiong Lee, Mohd Yahaya Norhamdan, Evan S. Vista, Wai Sin Chan, Sy Bui Cong, Pham Thi Thanh Tam, Jean-Marc Kaufman, Jean-Yves Reginster, Nicola Veronese, Ester Penserga

**Affiliations:** 1 Department of Medicine, Subang Jaya Medical Centre, Subang Jaya, Selangor Malaysia; 2 Department of Orthopaedics, Vinai Parkpian Orthopaedic Research Center, Faculty of Medicine, King Chulalongkorn Memorial Hospital, Thai Red Cross Society, Chulalongkorn University, Bangkok, 10330 Thailand; 3 College of Medicine De La Salle Medical Health Science Institute (DLSMHSI), Dasmariñas, Cavite Philippines; 4 Ageing and Age-Associated Disorders Research Group, Department of Medicine, Faculty of Medicine, University of Malaya, Kuala Lumpur, Malaysia; 5 Department of Medical Sciences, Faculty of Healthcare and Medical Sciences, Sunway University, Bandar Sunway, Malaysia; 6 Department of Medicine, Philippine General Hospital-University of the Philippines College of Medicine, Manila, Philippines; 7 Department of Orthopedic Surgery, Beacon Hospital, Petaling Jaya, Malaysia; 8 Department of Orthopaedics and Traumatology, Faculty of Medicine, Universiti Kebangsaan Malaysia, Kuala Lumpur, Malaysia; 9 Rheumatology, Allergy and Immunology Center, St. Luke’s Medical Center, Manila, Philippines; 10 Orthopaedic Surgeon of Orthopaedic Department of Centro Hospitalar Conde de Sao Januario, Macao, China; 11 Department of Rheumatology, 103 Military Hospital, Hanoi, Vietnam; 12 Rheumatology Department of 30-4 Hospital, Ho Chi Minh City, Vietnam; 13 Department of Endocrinology, State University of Ghent, Ghent, Belgium; 14 Public Health, Epidemiology and Health Economics Department, ULiège, Liège, Belgium; 15 Chair for Biomarkers of Chronic Diseases, Biochemistry Department, College of Science, King Saud University, Riyadh, Kingdom of Saudi Arabia; 16 Centre Académique de Recherche Et D’Expérimentation en Santé (CARES SPRL), Liège, Belgium; 17 Geriatric Unit, Department of Internal Medicine and Geriatrics, University of Palermo, V.le F. Scaduto 61, 90144 Palermo, Italy; 18 College of Medicine University of the Philippines, Manila, Philippines

**Keywords:** Knee osteoartrhitis, Patented crystalline glucosamine sulfate, Symptomatic slow-acting drugs for osteoarthritis, Algorithm

## Abstract

**Background:**

Since 2014, the European Society for Clinical and Economic Aspects of Osteoporosis, Osteoarthritis and Musculoskeletal Diseases (ESCEO) algorithm for the management of knee osteoarthritis (OA) is available worldwide.

**Aim:**

Based on this document, a Southeast Asia Working Group (SEAWG) wished to see how the new ESCEO algorithm developed in 2019 was perceived by Southeast Asian experts and how it was integrated into their clinical practice.

**Methods:**

A SEAWG was set up between members of the international ESCEO task force and a group of Southeast Asian experts.

**Results:**

Non-pharmacological management should always be combined with pharmacological management. In step 1, symptomatic slow-acting drugs for osteoarthritis are the main background therapy, for which high-quality evidence is available only for the formulations of patented crystalline glucosamine sulfate and chondroitin sulfate. In step 2, oral NSAIDs are a useful option, considering the cardiovascular/renal/gastrointestinal profiles of the individual patient. Intra-articular hyaluronic acid and corticosteroids are a possible alternative to oral NSAIDs, but limited evidence is available. If steps 1 and 2 do not give adequate relief of symptoms, tramadol can be used, but its safety is debated**.** In general, the indications of the ESCEO algorithm are important in Southeast Asian countries, but the reimbursement criteria of local health systems are an important aspect for adherence to the ESCEO algorithm.

**Conclusion:**

This guidance provides evidence-based and easy-to-follow advice on how to establish a treatment algorithm in knee OA, for practical implementation in clinical practice in Southeast Asian countries.

## Introduction

Osteoarthritis (OA) is one of the most common joint diseases in older people, mainly characterized by joint pain and stiffness with deep consequences on functional decline/disability and loss in quality of life [1, 2]. Knee OA is the most common localization within the symptomatic form affecting more than 250 million people worldwide [3]. Knee OA is ranked among the most common causes of global disability in terms of Disability-Adjusted Life Years (DALY) and poor quality of life [4–6].

In 2014, the European Society for Clinical and Economic Aspects of Osteoporosis, Osteoarthritis and Musculoskeletal Diseases (ESCEO) published some recommendations for the management of knee OA, developing a treatment algorithm that may give practical guidance for the prioritization of interventions and guiding physicians through progressive steps [7]. However, since the publication of the 2014 algorithm, new research has become available, with a particular attention to the safety of many medications commonly used to treat knee OA [8–11]. In 2016, as new observational data regarding drug safety became available, an update to the ESCEO algorithm was published [12].

Therefore, in 2019, a new algorithm [13] was published taking into account the recent evidence on efficacy and safety of medications commonly used for knee OA and the GRADE (Grading of Recommendations Assessment, Development and Evaluation) process was added, to better highlight the evidence used in the algorithm in a transparent and systematic way [14]. Since 2014, the ESCEO algorithm has been well-received worldwide and endorsed by many national societies with a consequent translation, adaptation to the local context, and publication in several countries including China, Russia, Central Europe and Southeast Asia [15–20]. For this reason, a working group (WG) was formed between members of the international ESCEO task force (N.V., J.M.K., and J.-Y.R.) and a group of Southeast Asian experts in knee OA (SEAWG) to see how Southeast Asian key opinion leaders perceive this algorithm and how it can be combined, with their own clinical practice, to harmonize and optimize the management of patients with knee OA throughout the world.

### Non-pharmacological treatment in the 2019 ESCEO algorithm

In the 2019 knee OA algorithm, non-pharmacological treatments (information/education; weight loss if overweight; and an exercise program mixing aerobic and strengthening exercises) have a major importance and is supported by a high level of evidence according to GRADE [7, 13, 21], even if the effect of these interventions is limited and their feasibility in the long term is still debated [22]. The Southeast Asian experts, during the workshop, highlighted the importance of non-pharmacological interventions (such as education and weight loss), including the use of Tai-Chi [23] and acupuncture [24] in patients in their clinical practice, even if this evidence for these last two interventions is supported by low quality studies.

## Pharmacological treatment in the 2019 ESCEO algorithm

### Step 1: background treatment

#### Paracetamol

Paracetamol (acetaminophen) is widely used for the treatment of knee OA symptoms, even though in 2014 ESCEO reported that this medication has only a small effect on pain and no significant effect on stiffness and physical function in patients with knee OA [25–27]. During 2014–2018, several concerns were available regarding safety over its routine chronic use, due to the increased risk of gastrointestinal (GI), cardiovascular (CV), hepatic and renal adverse events (AEs) [28] and increased mortality [29]. Surprisingly, even if paracetamol is widely used, its mechanism of action is not completely known, even if it is likely to involve cyclo‐oxygenase‐2 (COX‐2) inhibition, particularly when the cellular environment is low in arachidonic acid and peroxides such as in gastrointestinal and cardiovascular systems [30].

Based on this evidence (limited effect and increased risk of AEs), the 2019 ESCEO reccomends that paracetamol should be used only for short periods, as rescue medication when there is the inefficacy of the background therapy and at doses less than 3 g /day [13].

#### SYSADOAs

In both 2014 and 2019 versions of the ESCEO algorithm, Step 1 treatment of knee OA, recommends initiation of background therapy with long-term SYSADOAs (Symptomatic Slow-acting Drugs for Osteoarthritis) [7, 13], even if this class includes several products such as glucosamine, chondroitin, diacerein, and avocado soybean unsaponifiables (ASU), which are supported by varying degrees of clinical efficacy and safety data.

Glucosamine and chondroitin are natural compounds. Glucosamine hydrochloride (GHCl) is obtained by extraction processes and used as a nutraceutical or over-the counter (OTC) products. In contrast, glucosamine sulfate (GS) is a more sophisticated product, which can be obtained only by a proprietary semi-synthetic route and stabilization process and that is used only in the prescription drug product, i.e. patented crystalline glucosamine sulfate (pCGS) [31]. Unfortunately, multiple formulations of GS are available [32], both as prescription-grade products and OTC, with the latter having small/varying amounts of glucosamine. On the contrary, there is extensive literature to suggest that only pCGS, at least at 1,500 mg per day, is able to deliver consistently high glucosamine bioavailability and plasma concentration in humans, which would result in good clinical efficacy [33–40]. Conversely, GHCl and non-crystalline glucosamine sulfate products (usually consisting of GHCl with the addition of sodium sulfate to get a “sulfate” labelling) have consistently been shown to be ineffective in the treatment of knee OA [33, 35, 41–44]. A similar discussion can be applied to chondroitin sulfate [45–50].

Based on this scientific evidence, ESCEO specifically recommends the use of pCGS and long-acting chondroitin sulfate products (the latter of which are not available in Southeast Asia) in both versions of 2014 and 2019 of the algorithm [7, 13]. At the same time as the new ESCEO algorithm was released, another respected society, the Osteoarthritis Research Society International (OARSI) also updated their guidelines [51]. A working group recently conveyed by ESCEO examined the similarities and differences between these two guidelines and provided a narrative document to help guide health-care providers through the complexities of non-surgical management of knee OA (reference enclosed to be added) [52]. Whereas many similarities between the two guidelines were observed, ESCEO strongly supports the use of pCGS and chondroitin sulfate, whereas OARSI does not support their use. The main reason for this discrepancy is that the OARSI guidelines, which were mainly prepared in a US-centric perspective, do not recognize the concept of “pharmaceutical-grade” or “prescription-grade” SYSADOAs, such compounds being unavailable on the US soil [51][51].

The judgement of ESCEO is also based on the safety of SYSADOAs. Except for diacerein, several randomized placebo-controlled trials (RCTs) have demonstrated that SYSADOAs are not associated with any increased risk of AEs, both total and specific [11].

With regards to AEs, concerns have been raised regarding the safety of pCGS in patients with diabetes. Glucosamine, in fact, is an amino sugar that might lead to hyperglycemia and insulin resistance by over activating the hexosamine pathway [53]. However, it was already known that, once in plasma, glucosamine “does not go back to glucose”, but is directly catabolized and, therefore, no interference with glucose metabolism is expected [54]. This is also supported from the clinical trials’ data. At common doses used for OA treatment, pCGS showed no interference with glucose metabolism in normoglycemic subjects and in most subjects with hyperglycemia, impaired insulin sensitivity, pre-diabetes or diabetes [41, 55]. In addition, a meta-analysis on the effects of glucosamine on glucose metabolism found that glucosamine, at the usual oral doses used in knee OA patients, is well-tolerated by normal, diabetic, or pre-diabetic patients [56]. In the PROOF trial, a non-significant increase in glycated hemoglobin levels was found in overweight women who received pCGS during the follow-up period [57, 58]. Thus, the SEAWG recommends to advise caution at the start of treatment with glucosamine in diabetic patients [59].

#### Topical NSAIDs

At the last step of step 1 (background therapy), topical non-steroidal anti-inflammatory drugs (NSAIDS) may be added as cyclic therapy if the patient is still symptomatic. Whilst there is sufficient evidence that these medications are safe [8], their efficacy has been only demonstrated in short-term RCTs and, therefore, more data are needed for giving these medications for longer periods [13]. The most recent algorithm suggested that topical NSAIDs may be used in preference to oral NSAIDs, particularly in frail patients with knee OA, or prior to use of oral NSAIDs. The SEWG was in agreement with the use of topical NSAIDs for the control of persistent pain in knee OA.

### Step 2: advanced pharmacological treatment

If the patient is still suffering from pain or has important limitations in the activities of daily living, step 2 of the updated version of the algorithm will commence. Step 2 consists of two approaches.

The first approach is the use of oral NSAIDs. Based on the literature available, particularly regarding the safety of these medications [10], ESCEO, in the 2019 algorithm, makes a strong recommendation to the use of oral NSAIDs (selective or non-selective) as Step 2 therapy, but only if used intermittently and for as briefly as possible [13]. Moreover, the use of oral NSAIDs should be based on the patient risk profile, taking in consideration cardiovascular, renal, gastrointestinal co-morbidities [13]. Oral NSAIDs, in fact, should be not used in case of clinical forms of cardiovascular (e.g. decompensanted heart failure), renal (e.g. chronic renal failure with a creatinine clearance < 30 ml/min) or gastrointestinal (e.g. upper or lower gastrointestinal hemorrhages). When permitted it is important to remember that ESCEO recommended that all NSAIDs should be used at the lowest effective dose for the shortest period of time necessary to control pain [13]. Moreover, when using oral NSAIDs in older people, it is also important to consider the role of drug interaction in the development of adverse drug reaction. Drug-drug interactions (DDIs) in poly-therapy are one of the commonest causes of medication errors in geriatric medicine, with an estimated prevalence of 20–40% [60]. When talking about oral NSAIDs, a nice study reported that these medications may act on MAP kinase (MAPK) signal transduction pathway in the synovial membrane [61], a pathway that can be affected by the use of other medications [62].

The second part of step 2 consists of the use of intra-articular medications, i.e. hyaluronic acid and corticosteroids. For both these intra-articular products, there is weak evidence that supports the use of hyaluronic acid and corticosteroids in those who cannot take oral NSAIDs. The reasons for this decision are based on inconclusive efficacy, higher risk of AEs when compared to placebo and only having shor-term RCTs supporting the use of these drugs [9, 13].

The SEAWG agreed to the judicious use of NSAIDs for acute exacerbation of knee OA with inflammatory component, after considering the patient profile and co-morbid conditions with reference to gastrointestinal, cardiac and renal diseases. The dose of NSAIDs should be the lowest effective dose.

### Step 3: last pharmacological treatment

Last pharmacological options for the severely symptomatic patient are represented by short-term weak opioids. Tramadol may offer good analgesia in knee OA [63, 64], but a recent meta-analysis of the safety of oral opioids used in OA found an increased risk of gastrointestinal, central nervous system, and dermatological AEs compared with placebo [65]. For this reason, ESCEO gives only a weak recommendation to the use of short-term weak opioids in Step 3, as the last pharmacological option before surgery [13]. A similar evidence base is available for duloxetine [13].

The SEAWG concurs to the use of low dose weak opioids, such as tramadol, with the needed precaution for their known adverse events of nausea, somnolence and vomiting.

### Step 4: end-stage disease management and surgery

Total knee replacement (TKR) is appropriate when all previous interventions have failed, if the patient is still symptomatic, and, in particular, when a significant loss in quality of life is present [66–68]. However, for symptomatic patients in whom surgery is cotraindicated, the last pharmacological resort could be oral or transdermal opioids [69], which should be prescribed following the guidelines for use of opioid analgesics in the management of non-cancer pain [70].

The SEAWG adds that background physical therapy is to be continued for surgery-averse patients or those where surgery is contraindicated.

## Specificities of osteoarthritis management for Southeast Asia

As declared in the Introduction, the main objective of this paper is to find a consensus between experts from various South-East Asian countries and to offer a reference document which takes into account the national specificities and tries to be also as consensual as possible with the document published by one non-South East Asian respected society.

As a group of Southeast Asian experts in OA management, the SEAWG has carefully reviewed the ESCEO algorithm, including the most recent update of this year, considering it to be almost similar to the clinical practice pathways in several Southeast Asian countries. Therefore, as described in this paper, the WG endorses the principles of the ESCEO algorithm, reaching a consensus regarding recommendations for the stepwise multi-modal treatment of knee OA in Southeast Asia. With this work, of course, we cannot affirm to have included all the guidelines present in Southeast Asia countries and that these are in line to the ESCEO algorithm. Some national countries have indeed, significant differences in their guidelines compared to what is recommended in the ESCEO algorithm (e.g. the use of oral NSAIDs in the first and not in the second step of knee OA management), but that after an extensive discussion among the experts present at the meeting, we reached a formal consensus and the experts co-signing this paper agreed to the ESCEO recommendations.

However, it should be recognized that, in clinical practice, treatment should be based upon the individualized assessment of the patient, considering patients’ needs and preferences, the subjective interpretation of the evidence by the physician, and of course, subject to the local availability of a medication. In Southeast Asia, many countries acknowledge the definitive treatment for severe OA to be surgical in the form of TKR and prevention to avoid this late stage is advocated by at least delaying disease progression with SYSADOAs. Not all SYSADOAs are available at pharmacological doses and, in Southeast Asia, may be included only OTC preparations for which the efficacy is still not clarified. Local practices and cultural variations employ traditional healing remedies without large-scale controlled clinical trials. Complementary and alternative remedies in the form of endemic topical and aromatic preparations as well as physical manipulation (e.g. ayurveda or acupuncture) provides symptomatic relief among OA patient sufferers historically, even if limited data are available. These treatment modalities must be taken into consideration when planning out a comprehensive management approach for the patient. Adopting the ESCEO knee OA guidelines is a structured approached that is well-applicable in each of Southeast countries.

Another important point is that the criteria for reimbursement, as well as the organization of the local health care system, significantly vary across Southeast Asian countries and this should be taken into consideration when choosing treatment options. Moreover, in the same country, there may be differences between rural and urban areas, for example, in terms of the availability of different pharmacological agents. Furthermore, not all SYSADOAs are reimbursable by the public health care systems and, in some cases, even if reimbursed, it is only for limited periods. For this specific reason, the SEAWG wish to highlight the importance of health-economic studies that should be done locally to increase the rate of reimbursement of pharmacological agents that can be used to reduce OA progression. At this stage, the ESCEO algorithm is mainly based on the scientific and clinical evidence regarding efficacy and safety. [13] However, since the works regarding economic aspects are increasingly recognized as important, a future document including the health economics, with a specific application to the various South East Asian countries, could be an interesting work to be done.

In Southeast Asia, knee OA is a significant problem. For example, in the Philippines, almost 4 million people suffer from this medical condition, but unfortunately it is not considered among those chronic conditions that should be addressed by specific public health programs. For this reason, in 2017, a Philippine National OA Multidisciplinary Program was proposed to treat OA better. This multidisciplinary approach took into consideration the indications and the steps given by the 2019 ESCEO algorithm [13]. The application of the Philippine OA Program does not differ in practice from the steps given in the ESCEO at core step and step 1, even if paracetamol is given as background therapy at low doses, In step 2, the WG noted that not all oral or topical NSAIDs are reimbursed by the Philippines health care. Overall, the Philippine experts strongly believe in the concepts of the 2019 ESCEO algorithm, highlighting the importance of education of health care workers and specialty referral for the most appropriate management of knee OA.

In Thailand, a recent consensus (Thai Consensus Conference on Pharmacological Management of Knee OA 2019) was held. The minutes of this meeting are freely available at this website: https://www.thaihipknees.org/information/manual-and-proceedings-of-the-thai-consensus-conference-on-pharmacological-management-of-knee-oa-20192/. This consensus involved 69 experts in knee OA and was divided in two main parts, i.e. oral and non-oral/topical medications for knee OA management. Overall, a strong consensus was reached for the use of oral NSAIDs prescribed as the first-line drug for knee OA, even if they should only be used for a short-term period and intermittently, with caution, and the patient’s comorbid conditions taken into account, avoiding the concomitant use of paracetamol. This consensus recognized the importance of pCGS (and not OTC products) as first line/background therapy, which should be used continuously (if possible) and without any age restrictions. New research results were presented based on a real-life study using pCGS in Thailand. In the study, 250 Thai older people, with more than 50 years, with grade 2–3 KL knee OA were followed for 24 weeks and treated with 1500 mg/day of pCGS. The results of this study showed that pain scores, as measured by the visual analogue scale significantly improved after treatment with pCGS. In addition, this study also showed a positive effect of pCGS on quality of life (measured with short form 36) and physical performance, as measured with the timed up-and-go test. Despite the observational nature of these data, this study opens up the idea of a possible effect of pCGS on physical function and quality of life.

In Malaysia, knee pain is extremely common and often due to knee OA. In the Community Oriented Program for the Control of Rheumatic Diseases (COPCORD) survey, 14.4% complained of pain in the joints and/or musculoskeletal pain. The knee site was responsible for about two/thirds of all complaints pertaining to the joints, and more than half those examined with knee pain had clinical evidence of OA [71]. In this country, knee pain is a common cause of presentation to the general practitioner, with one study showing it to be the 7th most common complaint. Due to the high frequency of knee pain and presumed OA in the community, a Malaysian Clinical Practice Guideline for the Management of OA was first published in 2002, with a second edition in 2013. For the future, the main aim would be to get health economic data assessing the economic burden of OA, and the cost-effectiveness of treatment with SYSADOAs.

Finally, Macau has about 636,000 inhabitants, with a long-expected mean life expectancy (about 86 years). Therefore, knee OA is a common condition in this country, representing about 15–50% of all orthopedic consultations. In Macau, the 2019 ESCEO algorithm is followed in all its steps. Regarding GS, its use was started in 1990 and now is available in different forms. Importantly, the Macau experts believe that pCGS is highly effective for knee OA symptomatology and that most old people like a simple once-daily dosage, even if many products (with different costs and effect on knee OA symptoms) are available in this country.

## Conclusions

Knee OA is a signifcant problem in Southeast Asian countries, associated, similar to the rest of the world, with a high rate of disability and poor quality of life. In this paper, the WG has briefly summarized the reccomendations of the 2019 ESCEO algorithm and highlighted areas where it applies to clinical practice in Southeast Asia. Overall, the steps of the algorithm are followed and recognized as important, even if the different reimbursment criteria may change the way the algorithm is followed. This guidance provides evidence-based and easy-to-follow advice on how to establish a treatment algorithm in patients with knee OA, for practical implementation in the Southeast Asian countries' clinical practice.
